# A case-control study of mastitis: nasal carriage of *Staphylococcus aureus*

**DOI:** 10.1186/1471-2296-7-57

**Published:** 2006-10-11

**Authors:** Lisa H Amir, Suzanne M Garland, Judith Lumley

**Affiliations:** 1 Mother & Child Health Research, La Trobe University, Melbourne, Australia; 2 Department of Microbiology and Infectious Diseases, The Royal Women's Hospital, Melbourne, Australia

## Abstract

**Background:**

Mastitis is a common problem for breastfeeding women. Researchers have called for an investigation into the possible role of maternal nasal carriage of *S. aureus *in the causation of mastitis in breastfeeding women.

**Methods:**

The aim of the study was to investigate the role of maternal *S. aureus *nasal carriage in mastitis. Other factors such as infant nasal *S. aureus *carriage, nipple damage, maternal fatigue and oversupply of milk were also investigated. A case-control design was used. Women with mastitis (cases, n = 100) were recruited from two maternity hospitals in Melbourne, Australia (emergency departments, breastfeeding clinics and postnatal wards). Breastfeeding women without mastitis (controls, n = 99) were recruited from maternal and child health (community) centres and the rooms of a private obstetrician. Women completed a questionnaire and nasal specimens were collected from mother and baby and placed in charcoal transport medium. Women also collected a small sample of milk in a sterile jar.

**Results:**

There was no difference between nasal carriage of *S. aureus *in breastfeeding women with mastitis (42/98, 43%) and control women (45/98, 46%). However, significantly more infants of mothers with mastitis were nasal carriers of *S. aureus *(72/88, 82%) than controls (52/93, 56%). The association was strong (adjusted OR 3.23, 95%CI 1.30, 8.27) after adjustment for the following confounding factors: income, private health insurance, difficulty with breastfeeding, nipple damage and tight bra. There was also a strong association between nipple damage and mastitis (adjusted OR 9.34, 95%CI 2.99, 29.20).

**Conclusion:**

We found no association between maternal nasal carriage of *S. aureus *and mastitis, but nasal carriage in the infant was associated with breast infections. As in other studies of mastitis, we found a strong association between nipple damage and mastitis. Prevention of nipple damage is likely to reduce the incidence of infectious mastitis. Mothers need good advice about optimal attachment of the baby to the breast and access to skilled help in the early postpartum days and weeks.

## Background

Mastitis is a common problem for breastfeeding women [1-3] yet it is a poorly researched topic [4]. Mastitis may be a noninfective inflammation, resolving with heat and increased breast drainage [5,6], or an infective process which may lead to a breast abscess [7].

The most commonly isolated organism in lactating women with mastitis is *Staphylococcus aureus *(*S. aureus*): present in 32% to 44% of breast milk samples [8-11]. *S. aureus *is a commensal which may colonise the nostrils, axillae, vagina and pharynx of 30 to 50% of adults; as well as damaged skin, such as traumatised nipples of lactating women [12,13]. When *S. aureus *are present in the nostrils, they may act as a reservoir of *S. aureus *for clinical infections in the host or may facilitate spread to other people [14]. A review has concluded that four studies conducted in the 1990s found that *S. aureus *nasal carriers had a relative risk of 7.1 (95%CI 4.6, 11.0) of surgical-site infections, due to wound colonisation by the patient's endogenous flora [15].

Researchers have called for an investigation into the possible role of maternal nasal carriage of *S. aureus *in the causation of mastitis [16]. In clinical practice, there is anecdotal evidence that maternal or infant nasal carriage may be linked to recurrent mastitis in lactating women [17]. Our literature review did not identify any studies investigating the role of maternal or infant nasal carriage of *S. aureus *in mastitis. Medline searches (via PubMed) were conducted using keywords "staph* AND (lactation OR breastfeeding OR postpartum OR mastitis)" limited to human studies. A recent update (31 May 2006) has identified one case study of a mother of premature quadruplets who had symptoms of mastitis; methicillin-resistant *S. aureus *(MRSA) was isolated in expressed breast milk and nasal cultures from mother and infants [18].

We conducted a case-control study to examine the possible role of maternal nasal carriage of *S. aureus *in the development of mastitis. The role of *S. aureus *nasal carriage in the infant and other factors reputed to predispose women to mastitis, such as nipple damage, maternal fatigue and oversupply of milk, were also assessed.

## Method

Cases were women with mastitis attending the Emergency Department or the Breastfeeding Clinic at the Royal Women's Hospital or Mercy Hospital for Women in Melbourne, Australia. Mastitis was defined as at least two breast signs or symptoms (pain, redness or lump) and one systemic symptom (fever or 'flu-like symptoms) present for at least twelve hours.

Women in the control group were lactating women (with babies aged six weeks or less) attending Maternal and Child Health (MCH) centres in metropolitan Melbourne (community clinics attended by new mothers). Also, women attending a private obstetrician for their six-week postnatal visit were invited to join the study.

After women gave written consent, they completed a questionnaire about nipple damage, oversupply of milk and other factors identified as possible predisposing factors for mastitis. Maternal fatigue was assessed using the four Vitality questions from the SF-36 [19]. (The SF-36 Health Survey is one of the most widely used health-related quality of life questionnaires, measuring eight health concepts, including Physical functioning, Social functioning and Vitality) [19]. At the end of the questionnaire for women with mastitis, participants were asked to describe "how you have been feeling and how mastitis has affected you"; the results of these open-text comments have been published separately [20].

Nasal specimens were collected from mother and baby. Saline-moistened swabs were rotated around the inside of the nasal vestibule, then placed in Amies charcoal transport medium. Women used a sterile water wipe to cleanse her nipple then expressed a small sample of milk in a sterile jar. Specimens were labelled and delivered to the microbiology laboratory at the Royal Children's Hospital, where the swabs were inoculated on Mannitol Salt Agar plates, a medium selective for staphylococci. An aliquot of expressed breast milk was also placed on a Mannitol Salt Agar plate. *S. aureus *was confirmed with DNase test (thermostable nuclease) and antibiotic susceptibility was conducted using standard microbiological methods.

Using Epi-Info 6.0 for an unmatched case-control study with 95% confidence and 80% power, if 20% of controls were nasal carriers and 40% of cases, we would need 91 women in each group. We planned to recruit 100 cases (women with mastitis) and 100 controls (women in the community). The data were analysed by Stata 8.0 computer program. The comparability of cases and control groups were described. Odds Ratios were calculated to compare exposures in each group, and Mantel-Haenszel Weighted Odds Ratios where appropriate. Logistic regression was used to determine factors predictive of mastitis.

Four infants over 6 weeks age were inadvertently recruited for the control group (two at 7 weeks, one 9 and one 11 weeks). A sensitivity analysis was conducted by repeating the analysis without these infants and the results were found to be almost identical: therefore the records of these four mothers and babies were retained in the sample.

Ethics approval:

• La Trobe University Human Ethics Committee (21/8/02 Project 02-61)

• Research and Ethics Committees, Royal Women's Hospital (23/7/02, Project 02/22)

• Research Ethics Committee, Mercy Hospital for Women (17/7/02, Project R02/32)

• Department of Human Services, Victoria (5/6/02, Project 36/02).

## Results

One hundred women with mastitis (cases) and ninety-nine breastfeeding women (controls) were recruited between August 2002 and April 2004. Recruiting was stopped as the database incorrectly indicated that 100 controls had been recruited.

Fifty-four women with mastitis were recruited at the Mercy Hospital for Women and 46 women at the Royal Women's Hospital; from the breastfeeding clinics (n = 38), readmitted with mastitis in the wards (n = 32) and through the emergency departments (n = 25; 5 missing data). Most of the women for the control group were recruited through MCH centres (n = 70), whilst 29 women were from the private obstetrician.

### Characteristics of study population

The background characteristics are displayed in Table 1. Women with mastitis were older than women in the control group (34 years and 32 years, p < 0.05), while the median age of the babies was 16 days for cases and 36 days for controls. Family income was lower in the women with mastitis and they were less likely to have private health insurance.

**Table 1:** Background characteristics of participants in a case control study of mastitis.

	**Cases (N = 100) n (*%*)**	**Controls (N = 99) n *(%)***	**Odds Ratio**	**95% CI**		Significance*
Mother's age (mean, years)	33.5	31.7				**p = 0.004
Mother's education:						
Completed secondary	89 *(89)*	90 *(91)*	1.24	0.49	3.13	p = 0.655
Completed tertiary degree	63 *(63)*	70 *(71)*	1.02	0.90	1.16	p = 0.779
Family income (n = 184)						
up to $50,000	20/92 *(22)*	10/92 *(11)*				
$50,001–70,000	29/92 *(32)*	17/92 *(19)*	0.85	0.32	2.24	
more than $70,000	43/92 *(47)*	65/92 *(71)*	0.33	0.14	0.77	**p = 0.004
Mother paid work/study	7 *(7)*	6 *(6)*	1.20	0.50	2.86	p = 0.687
Private health insurance	51 (51)	78 (80)	0.27	0.14	0.50	**p = 0.000
Baby's age in days						
(mean and range)	33.7 (6–728)	34 (13–77)				t-test ns
median	16	36				
Multiple birth (twins)	3 *(3)*	3 *(3)*	0.99	0.19	5.03	p = 0.990
Primiparity	69 *(69)*	60 *(61)*	0.69	0.39	1.24	p = 0.216
Caesarean section	35 *(35)*	32 *(32)*	1.13	0.63	2.03	p = 0.690

(n = 100 women with mastitis and 99 breastfeeding controls). Melbourne, Australia, 2002–2004.

*chi-square test for all analyses, except t-test for mother's and baby's age.

**statistically significant.

ns = not significant.

Table 2 shows the health of women and their infants. After a question to determine if women had experienced mastitis in the past, women were asked "Have you ever had any other staph bacterial infections? For example: boils, abscesses, sores inside your nose?" Women with mastitis were more likely to have self-reported a past history of staphylococcal infection(s), 23% compared to 12%.

**Table 2:** Health characteristics of participants in a case control study of mastitis.

	**Cases (N = 100) n *(%)***	**Controls (N = 99) n *(%)***	**Odds Ratio**	**95% CI**		Significance*
**Mother's Health**						
Breast surgery:	7/100 *(7)*	5/99 *(5)*	1.42	0.43	4.62	p = 0.565
Past history staphylococcal infection	23/100 *(23)*	12/99 *(12)*	2.17	1.01	4.64	**p = 0.047
Past history candida infection	16/97 *(17)*	8/95 *(9)*	2.15	0.87	5.29	p = 0.096
Past history mastitis (previous child)	12/100 *(12)*	12/99 *(12)*	0.99	0.42	2.32	p = 0.979
Smoker	5/100 *(5)*	4/99 *(4)*	1.25	0.33	4.80	p = 0.745
Anaemic	10/100 *(10)*	5/99 *(5)*	2.09	0.69	6.35	p = 0.194
Scored below average on vitality score (from SF36)	61/100 *(61)*	26/99 *(26)*	4.39	2.41	8.01	**p = 0.000

**Baby's Health**						
Unwell	16/100 *(16)*	11/98 *(11)*	1.51	0.66	3.43	p = 0.330
Difficulty breastfeeding	57/100 *(57)*	14/99 *(14)*	8.05	4.04	16.05	**p = 0.000

(n = 100 women with mastitis and 99 breastfeeding controls). Melbourne, Australia, 2002–2004.

*chi-square test for all analyses.

**statistically significant.

The sum of the scores from the four Vitality questions in the SF36 ranged from 4 (low) to 17 (high), with a mean of 10.7 (n = 195). Women were dichotomised into two groups scoring above or below the mean, and the groups compared. Sixty-one percent of women with mastitis scored below the mean vitality score, compared to 26% of controls. However, it is possible that some women were reporting lethargy associated with the onset of mastitis.

The breastfeeding characteristics of the sample can be seen in Table 3. Cases were more likely to have had nipple pain (71%) compared to controls (35%), and more likely to be using purified lanolin on their nipples (64%) than controls (35%). Thirteen cases used topical antifungal cream/ointment/gel, while nine controls used topical antifungal treatment (not significant). More cases used nipple shields (16%) than controls (2%). Cases were also more likely to have experienced breast engorgement in the previous week (51%) than controls (19%), were more likely to say that they had missed a feed (54%) than the controls (36%) and that they had too much milk: 29% compared to 17%. Women were asked about pressure on their breasts in the previous week. More cases reported pressure from a tight bra (37%) than controls (20%).

**Table 3:** Breastfeeding characteristics of participants in a case control study of mastitis.

	**Cases (N = 100)** **n *(%)***	**Controls (N = 99)** **n *(%)***	**Odds Ratio**	**95% CI**		Significance*
Nipple pain	70/99 *(71)*	35/99 *(35)*	4.41	2.43	8.02	*p = 0.000
Grazed nipple	47/99 *(48)*	9/99 *(9)*	9.04	4.10	19.93	*p = 0.000
Cracked nipple	44/99 *(44)*	5/99 *(5)*	15.04	5.63	40.19	*p = 0.000
White spot	16/98 *(16)*	7/99 *(7)*	2.56	1.01	6.54	*p = 0.049
Breast engorgement (needed to express)	51/100 *(51)*	19/99 *(19)*	1.76	1.19	2.61	*p = 0.005
Blocked duct	52/100 *(52)*	13/99 *(13)*	3.70	2.39	5.71	*p = 0.000
Missed feeds	53/99 *(54)*	36/99 *(36)*	1.76	1.05	2.93	*p = 0.032
More than 6 hours between feeds	45/100 *(45)*	38/99 *(38)*	1.31	0.75	2.31	p = 0.344
Hurrying feeds	38/100 *(38)*	48/99 *(48)*	0.92	0.55	1.54	p = 0.745
Feeding less than usual	11/98 *(11)*	4/99 *(4)*	0.95	0.70	1.31	p = 0.770
Pressure on breast: bra	37/99 *(37)*	20/99 *(20)*	2.36	1.25	4.46	*p = 0.008
Thickness of milk	8/98 *(8)*	1/99 *(1)*	0.95	0.62	1.47	p = 0.833
Too much milk	29/100 *(29)*	17/99 *(17)*	1.37	1.09	1.73	*p = 0.007
Baby prefers one breast	48/100 *(48)*	37/99 *(37)*	1.55	0.88	2.72	p = 0.1293
Amount of breastfeeding:						
Fully	77/100 *(77)*	84/99 *(85)*				
Breast and formula	13/100 *(13)*	15/99 *(15)*				
Expressing only	10/100 *(10)*	0/99 *(0)*				*p = 0.005
Lanolin on nipples	64/100 *(64)*	35/99 *(35)*	3.25	1.82	5.81	*p = 0.000
Nipple shields	16/100 *(16)*	2/99 *(2)*	9.24	2.06	41.35	*p = 0.004
Breast pads	76/100 *(76)*	85/99 *(86)*	0.52	0.25	1.08	p = 0.080
Expressing	86/100 *(86)*	51/99 *(51)*	5.78	2.90	11.51	*p = 0.000
Always washed nipples	1/100 *(1)*	1/99 *(1)*	0.99	0.06	16.05	p = 0.994
Always washed hands	48/100 *(48)*	41/99 *(41)*	1.31	0.75	2.29	p = 0.351

(n = 100 women with mastitis and 99 breastfeeding controls). Melbourne, Australia, 2002–2004.

*chi-square test for all analyses.

**statistically significant.

Women with mastitis were more likely to report that their infant was having difficulty with breastfeeding, 57%, than the control group, 14%. Ten women with mastitis were currently feeding their infant expressed breast milk only, while none of the women in the control group was expressing only.

### Microbiological results

There was no difference between nasal carriage of *S. aureus *in women with mastitis (43%) and women in the control group (46%) (Table 4). The overall proportion of women with a positive nasal culture for *S. aureus *was 44.4% (Binomial Exact 95% CI 37.3, 51.6). (MRSA was not isolated in any specimens in this study).

**Table 4:** *S. aureus *results of a case control study of mastitis.

	**Cases (N = 100)** **n (%)**	**Controls (N = 99)** **n (%)**	**Odds Ratio**	**95% CI**		Significance*
**Mother**						
Nasal swab positive for *S. aureus*	42/98 *(43)*	45/98 *(46)*	0.88	0.50	1.55	p = 0.666
Sparse	23	15				
Moderate	10	21				
Profuse	8	8				p = 0.0614
Expressed breast milk positive for *S. aureus*	45/99 *(46)*	15/86 *(17)*	3.94	1.99	7.81	**p = 0.000
Sparse	17	11				
Moderate	14	3				
Profuse	14	1				**p = 0.0339

**Baby**						
Nasal swab positive for *S. aureus*	72/88 *(82)*	52/93 *(56)*	3.55	1.80	7.00	**p = 0.000
Sparse	9	6				
Moderate	15	20				
Profuse	47	26				p = 0.1053

(n = 100 women with mastitis and 99 breastfeeding controls). Melbourne, Australia, 2002–2004.

*chi-square test for all analyses.

**statistically significant.

As expected, the expressed breast milk of women with mastitis was more likely to be positive for *S. aureus *(45/99, 46%), than the milk of controls (15/83, 18%). Most of the *S. aureus *isolated from the milk of the controls was reported as 'sparse" (11/15, 73%). Only one specimen in the control group was reported as "profuse" (1/15, 7%) compared to 14/45 (31%) in the mastitis group.

Significantly more infants of mothers with mastitis were nasal carriers of *S. aureus *(72/88, 82%) than infants in the control group (52/93, 56%, OR 3.55, 95%CI 1.80, 7.00). A high proportion of *S. aureus *in both groups was reported as "profuse", 66% of cases and 50% of controls, 59% in total. Overall, 68.5% of infants were nasal carriers of *S. aureus *(Binomial Exact 95% CI 61.2, 75.2).

The youngest infants were most likely to be nasal carriers (91% of infants in the first two weeks in the mastitis group), compared to 78% of infants aged 5–6 weeks. A stratified analysis of *S. aureus *nasal carriage in the infants was conducted to examine the results in babies at different ages. The Mantel-Haenszel weighted Odds Ratio was 3.49 (95%CI 1.38, 8.83) for infants of mothers with mastitis to be nasal carriers compared to infants in the control group (Table 5).

**Table 5:** Babies' nasal results of a case control study of mastitis.

	**Cases N *(%)***		**Controls N *(%)***				
	***S. aureus *pos**	***S. aureus *neg**	***S. aureus *pos**	***S. aureus *neg**	**Odds Ratio**	**95% CI**	

**Babies 1–2 w**	41 *(91)*	4	2 *(67)*	1	6.67	0.08	156.3
**Babies 3–4 w**	22 *(82)*	5	17 *(53)*	15	3.71	0.99	15.45
**Babies 5–6 w**	7 *(78)*	2	33 *(59)*	23	2.52	0.42	26.64
**Babies >6 w**	2 *(29)*	5	0 *(0)*	2			
**Total**	72 *(82)*	16	52 *(56)*	41	3.49*	1.38	8.83

(n = 88 infants of mothers with mastitis and 93 control infants), Melbourne, Australia, 2002–2004: stratified by age.

*Mantel-Haenszel Weighted Odds Ratio.

w = week(s).

A statistically significant association was found between women with a cracked nipple and nasal carriage of *S. aureus *in their infants. Eighty-four percent (38/45) of women with a cracked nipple had a baby with nasal *S. aureus*, compared to 63% (85/135) of women without a cracked nipple, OR 3.19 (95%CI 1.33, 7.69). However, there was no association between nasal carriage in the mother and a cracked nipple: carriage in women with a cracked nipple was 39% (19/49) compared to 46% (67/146) of women without a cracked nipple.

### Multivariate analysis

A logistic regression model was developed to look at factors predictive of mastitis. The independent variables of interest were tested individually against the dependent variable and were entered in the model if the p-value of the Wald statistic was ≤ 0.25 [[21], p95]. Where there were small numbers of missing values, records were deleted (seven records). Fifteen women had missing values for income, and sixteen babies did not have a result for nasal swab. These records were retained with the missing values coded accordingly. This left 192 records for analysis.

The initial model included the following variables: mother's age, income (2 levels), private health insurance, past history of staphylococcal infection, baby having difficulty with breastfeeding, nipple cracked, engorged breast/s, missed feed/s, tight bra, too much milk, using lanolin on nipple/s, baby positive for nasal *S. aureus*, mother anaemic, baby prefers one breast. Variables were eliminated one at a time using logistic regression. Only those with a p-value of the Wald statistic ≤ 0.05 were retained in the model. The process was repeated until only significant variables remained. Then all independent variables eliminated in the original univariate analysis were added back into the model one at a time to check that none was now significant given the reduced model. The lroc test identified that the area under ROC curve was 0.8778, that is a high sensitivity, and the lstat test showed 80.73% correctly classified. The final model (Model 1) is presented in Table 6.

**Table 6:** Logistic regression of a case control study of mastitis.

	**Univariate analysis**			**Multivariate analysis Model 1 (n = 192)**				**Multivariate analysis Model 2 (n = 192) (No demographic variables)**			
	**Odds Ratio**	**(95% CI)**		**Odds Ratio**	**(95% CI)**		**P**	**Odds Ratio**	**(95% CI)**		**P**

Income											
<Aus$70,000	Reference			Reference				-	-	-	-
>Aus$70,001	0.37	0.20	0.68	0.31	0.13	0.71	0.006	-	-	-	-
Missing	0.67	0.22	2.06	0.28	0.06	1.14	0.120	-	-	-	-
PHI											
No	Reference			Reference				-	-	-	-
Yes	0.27	0.14	0.51	0.23	0.10	0.55	0.001	-	-	-	-
Difficulty breastfeeding											
No	Reference			Reference				Reference			
Yes	7.76	3.86	15.58	6.32	2.53	15.76	0.000	4.64	2.11	10.22	0.001
Cracked nipple											
No	Reference			Reference				Reference			
Yes	15.02	5.60	40.33	9.34	2.99	29.20	0.000	9.39	3.24	27.21	0.000
Tight bra											
No	v			Reference				Reference			
Yes	2.31	1.21	4.41	3.47	1.30	9.22	0.013	2.38	1.06	5.36	0.036
Baby nasal *S. aureus*											
No	Reference			Reference				Reference			
Yes	3.70	1.85	7.40	3.23	1.30	8.27	0.017	2.70	1.19	6.14	0.018
Missing	4.56	1.41	14.71	3.49	0.78	15.50	0.101	3.20	0.83	12.38	0.092

(n = 98 women with mastitis and 94 breastfeeding controls). Melbourne, Australia, 2002–2004. Model 1 includes income, private health insurance, difficulty with breastfeeding, damaged nipple, tight bra, baby nasal S. aureus, and Model 2 includes difficulty with breastfeeding, damaged nipple, tight bra and baby nasal S. aureus.

The adjusted Odds Ratio for infants of mothers with mastitis to be nasal carriers was 3.23 (95%CI 1.30, 8.27) after adjusting for possible confounding factors (Model 1). In order to explore the effects of breastfeeding factors and baby nasal carriage without including demographic factors, a second model was developed (Model 2). Without including the demographic variables (income and private health insurance), the second model is very similar to Model 1. In a third model (not shown), the demographic variables were included while limiting the analysis to private patients (n = 126), and the results were also similar. A fourth model (not shown, n = 184), excluding all babies over 7 weeks also found that infant nasal carriage was significant (adjusted OR 4.08, 95%CI 1.44, 11.67).

## Discussion

### Summary of main findings and comparison with existing literature

The study showed that there was no difference in the proportion of women with mastitis and without mastitis who were nasal carriers (43% and 46% respectively). The overall proportion of women with a positive nasal culture for *S. aureus *was 44.4% (95% CI 37.3, 51.6). This is consistent with the mean of 37.2% in general populations calculated by Kluytmans and colleagues from eighteen studies in 13,873 people [15], but seems higher than other studies published in 2004: 29% [22], 24% [23] and 33% [24].

We found that a very high proportion of infants of mothers with mastitis were nasal carriers: 82% and this was statistically significantly higher than infants of other women (56%). Infant nasal carriage remained significant after adjusting for other variables. Younger infants were most likely to be nasal carriers than older infants in this study. In a similar manner, Peacock and colleagues found that 40–50% of infants were colonised with *S. aureus *in the first eight weeks, falling to 21% by six months [25].

An association was not found between parity or a history of mastitis and being a case in this study, in contrast to previous studies [2,16]. This may be related to the high proportion of primiparous women in both groups of our study. Also, women with a history of mastitis may have been more likely to volunteer to be a control than other women.

The presence of a cracked nipple was associated with a high odds for mastitis, 9.34 (95%CI 2.99, 29.20), after adjusting for other factors. Foxman and colleagues also found an association with "nipple cracks or sores" with an OR of 3.4 (95%CI 2.04, 5.51) on logistic regression [16]. Prevention of nipple damage is likely to reduce the incidence of infectious mastitis. New mothers need good advice about optimal attachment of the baby to the breast and access to skilled help in the early postpartum days and weeks.

This study found that using lanolin on nipples was significantly associated with mastitis on univariate analysis; however this was no longer significant on multivariate analysis. We expect that the association between creams on nipples and mastitis [26] is more likely to be related to the fact that nipple creams tend to be used when the nipples are damaged, and it is the nipple damage that probably is the route by which infection enters the breast rather than the creams themselves.

*S. aureus *was isolated in the milk of 46% of women with mastitis, a similar proportion to studies over the last thirty years [11]. *S. aureus *was also isolated in the milk of 17% of women without mastitis; mostly reported as "sparse" (11/15), probably reflecting contamination of the milk by bacteria on the skin of the nipple or the hands. In other studies between 0 and 20% of milk specimens from healthy women are positive for *S. aureus *[27,28].

Although it is not possible to conclude whether transmission occurred from the infant to the mother's breast or visa versa, it is likely that *S. aureus *was transmitted from the infant to the mother. In 1957, Wysham and colleagues demonstrated that 7 of 9 infants with positive throat cultures for *S. aureus *transmitted the organism to their bottle of formula milk [29]. Babies are born sterile and acquire their colonisation from their mother or the hands of health workers. Mothers and infants have been shown to be likely to carry the same strain of *S. aureus *[25,30]. Staphylococci may be transferred from the mother's nose to the infant's and then back to the mother's nipple, particularly if the nipple has been traumatised.

### Strengths and limitations of this study

The diagnosis of mastitis relied on clinical signs and symptoms as there are no definitive tests for mastitis in women. The women in this study experienced either fever or systemic symptoms for at least 12 hours. Future studies could assess the usefulness of testing milk for the presence of leukocytes [31]. Molecular microbiology (eg pulsed field gel electrophoresis, PFGE) testing of isolates could have confirmed the clonality of *S. aureus *strains present in mothers with mastitis and in their infants. However, funding for this study was not sufficient to conduct PFGE.

It was originally planned to recruit women for the control group through MCH centres in the community (n = 25). However, we relied on women being referred to the study and recruitment was slow, so we started recruiting women attending a private obstetrician for their six-week postnatal check up. It would have been inappropriate to recruit women attending the hospital (Emergency Department or Breastfeeding clinic) as we were seeking women without problems for the control group. Therefore the controls were more likely to be private patients than cases, which resulted in more women from the higher income group as controls than cases. In order to assess if this had an effect on the study results, a logistic regression model was developed using only women with private insurance and similar results were obtained as when the model included all women.

A limitation of the case-control design is that any associations identified cannot be concluded to be causal. For example, private health insurance appears to be protective against mastitis (OR 0.27, 95%CI 0.14, 0.51), but this association is due to the selection bias that occurred during recruitment. The association between nasal *S. aureus *carriage in infants and mastitis in their mothers (adjusted OR 3.19, 1.23, 8.29) appears to be robust as it was significant in each logistic regression model. However, it does not tell us if this association proves a link between nasal carriage and mastitis nor in which direction the transmission is occurring.

### Implications for clinical practice and future research

Mastitis is an acute painful illness, not limited to the breast, and often associated with a negative emotional response [20]. In order to prevent mastitis, clinicians could advice new mothers about the factors commonly associated with this problem, such as milk stasis caused by missed feeds, expressing and breast restriction [32]. Breastfeeding women can be alert for the early symptoms of mastitis when they have been extra busy, for example when travelling or when they have visitors staying. If women have anticipatory guidance they can overcome milk stasis in these situations by increasing breastfeeds or expressing more frequently.

Future studies need to aim to collect clinical specimens prospectively in order to determine the transmission dynamics between mother and infant. Molecular microbiology (e.g. PFGE) can be used to confirm that the same strain of *S. aureus *is present in mother and child, and the direction of transmission of organisms between mother and child.

It is not standard practice for mothers to wash hands before breastfeeding (less than 50% of women in both groups "always" washed hands). Future studies could focus on hand washing as *S. aureus *may be carried transiently on the hands [33] and can then be transferred to the breast. Hospitals should be aware of the possibility of transmission of potential pathogens on breast pumps; disinfection is particularly important after equipment is used by women with breast infections.

Another topic for future research is recurrent mastitis. Would it be possible to reduce recurrences of mastitis by reducing nasal carriage of *S. aureus *in mothers and infants where mothers have already experienced an episode of mastitis?

## Conclusion

In conclusion, we found no association between maternal nasal carriage of *S. aureus *and mastitis, but nasal carriage in the infant was associated with breast infections. As in other studies of mastitis, we found a strong association between nipple damage and mastitis.

## Competing interests

The author(s) declare that they have no competing interests.

## Authors' contributions

The study was conducted by LHA as part of her PhD. SMG and JL supervised the project and contributed to the study design, analysis and writing.

## Pre-publication history

The pre-publication history for this paper can be accessed here:


